# Cytochrome P450 2E1 predicts liver functional recovery from donation after circulatory death using air-ventilated normothermic machine perfusion

**DOI:** 10.1038/s41598-022-11434-y

**Published:** 2022-05-06

**Authors:** Ji-Hua Shi, Dong-Jing Yang, Qiang Jin, Nuo Cheng, Yuan-Bin Shi, Yang Bai, Dong-Sheng Yu, Wen-Zhi Guo, Guang-Bo Ge, Shui-Jun Zhang

**Affiliations:** 1 Department of Hepatobiliary and Pancreatic Surgery, Henan Key Laboratory of Digestive Organ Transplantation and Zhengzhou Key Laboratory for HPB Diseases and Organ Transplantation, The First Affiliated Hospital of Zhengzhou University, Zhengzhou University, Zhengzhou, China; 2 Institute of Interdisciplinary Integrative Medicine Research, Shanghai University of Traditional Chinese Medicine, Shanghai, 200473 China; 3 Division of Pharmacology, The First Affiliated Hospital of Zhengzhou University, Zhengzhou University, Zhengzhou, China

**Keywords:** Predictive markers, Hepatology, Molecular medicine

## Abstract

The optimal oxygen concentration is unclear for normothermic machine perfusion (NMP) of livers from donation after circulatory death (DCD). Our purposes were to investigate the effect of air-ventilated NMP on the DCD liver, analyze the underlying mechanism and select the targets to predict liver functional recovery with NMP. NMP was performed using the NMP system with either air ventilation or oxygen ventilation for 2 h in the rat liver following warm ischemia and cold-storage preservation. *Proteomics* *and metabolomics* were used to reveal the significant molecular networks. The bioinformation analysis was validated by administering peroxisome proliferator activator receptor-γ (PPARγ) antagonist and agonist via perfusion circuit in the air-ventilated NMP. Results showed that air-ventilated NMP conferred a better functional recovery and a less inflammatory response in the rat DCD liver; integrated *proteomics* *and* *metabolomics* analysis indicated that intrahepatic docosapentaenoic acid downregulation and upregulation of cytochrome P450 2E1 (CYP2E1) expression and activity were associated with DCD liver functional recovery with air-ventilated NMP; PPARγ antagonist worsened liver function under air-oxygenated NMP whereas PPARγ agonist played the opposite role. In conclusion, air-ventilated NMP confers a better liver function from DCD rats through the DAP-PPARγ-CYP2E1 axis; CYP2E1 activity provides a biomarker of liver functional recovery from DCD.

## Introduction

Normothermic machine perfusion (NMP) could deliver oxygen and recreate the physiological environment of oxygenation and cellular metabolism. This might minimize liver ischemia followed by reperfusion injury, help to estimate liver graft functionality and benefit functional recovery before graft implantation, especially for organs from donation after circulatory death (DCD)^1,2^. Thus, NMP has theoretical advantages over static cold storage (SCS) in benefiting liver transplantation by utilization of DCD donors. Animal transplant models and clinical data have suggested that the use of NMP might improve organ utilization and posttransplant outcomes^3^. However, few sensitive parameters have been developed to predict liver functional recovery from DCD liver with NMP. The reason is partly due to that the mechanisms underlying the benefits of NMP are not completely understood.

The most commonly used approach for applying NMP in clinics is the replacement of cold preservation prior to graft implantation. Experimental models have demonstrated that warm and cold liver ischemia followed by reperfusion injury, that is named ischemia–reperfusion injury, is characterized by oxidative tissue injury and activation of the inflammatory immune response either in situ at implantation or during NMP^4,5^. Initial dysfunction in mitochondria and endoplasmic reticulum from warm ischemia from DCD may induce a lower threshold of reperfusion injury in liver grafts compared with donors after brain death^6^. Our previous study demonstrated that endoplasmic reticulum stress is highly activated during liver NMP from DCD rats, and that administration of acting transcript factor 6 agonist under NMP could improve liver function^7^.

Constant oxygenation during machine perfusion is of special interest in the context of graft functional recovery. Oxygen in the perfusate may help the fast recovery of mitochondrial function following liver cold preservation. Meanwhile, the availability of oxygen, especially at high concentrations of oxygen may foster formation of reactive oxygen species (ROS). This is of even greater concern under re-oxygenation conditions where the perfusion ROS-mediated injury occurs. This ROS-mediated injury potentially restricts organ function after transplantation. Against this background, different concentrations of oxygen draw attention to yield the positive effects of active oxygenation while avoiding adverse effects^8^. Application of hypothermic machine perfusion (HMP) 100% oxygen for kidney grafts from DCD resulted in a better function compared to HMP with no oxygen or air^9^. So far, it remains unclear what oxygen concentration is optimal for NMP.

Our previous study demonstrated the arterial oxygen-ventilated NMP system could improve function of DCD liver using the rat model^7^. Through our pilot experiment based on the established rat model^7^, arterial oxygen-ventilated NMP system could create an oxygen pressure of over 400 mmHg in the artery (PaO_2_ > 400 mmHg), which agrees with the most reported NMP systems^4^, whereas arterial air-ventilated NMP could produce near-to-physiological oxygenation (PaO_2_ = 90~200 mmHg). Thus, our primary purpose in the current study was to compare the effect of air-ventilated NMP with oxygen-ventilated NMP on liver function from DCD rats before implantation. Based on the findings, the further purpose was to investigate the mechanism of liver functional recovery using an arterial air-ventilated NMP system on liver function through an integrative *proteomics* *and* *metabolomics* analysis, and select the suitable target to predict liver functional recovery from DCD liver with NMP.

## Methods

### Animal and study design

The animal experiment was approved by the Animal Ethics Committee of Zhengzhou University, Zhengzhou, China (No.2019-KY-019). All methods were carried out in accordance with relevant guidelines and regulations along with the ARRIVE (Animal Research: Reporting of In Vivo Experiments) guidelines statement. The Sprague–Dawley male rats weighing 320–350 g were purchased from Beijing Vital River Laboratory Animal Technology Co., Ltd. (Beijing, China). All rats were housed under specific pathogen-free conditions at room temperature with a 12/12 h light/dark cycle, and were allowed free access to chow and water before surgical procedures.

Based on the accepted concept that oxygenated NMP would benefit functional recovery of liver graft^1,3^, we designed the experimental groups (Table 1) to compare the effects of near-to-physiological oxygenation (PaO_2_: 90~200 mmHg) and hyperoxygenation (PaO_2_ > 400 mm Hg) of the perfusate on liver graft: ① Group control (n = 6), healthy livers without ischemia; ② Group hyperoxygenated NMP (n = 6), DCD livers exposed to 30 min of in situ warm ischemia and 8 h cold ischemia preservation followed by 2 h NMP with oxygen ventilation; and ③ Group air-oxygenated NMP (n = 6), DCD livers exposed to 30 min of in situ warm ischemia and 8 h cold ischemia preservation followed by 2 h NMP with air ventilation.

**Table 1:** Design of animal experiment (N = 30).

Group		Harvested liver	Normothermic machine perfusion (NMP)	Perfusate
Control	N = 6	Healthy livers without ischemia	–	–
Hyperoxygenated NMP	N = 6	30 min of in situ warm ischemia followed by 8 h cold ischemia preservation	2 h NMP with oxygen ventilation	–
Air-oxygenated NMP	N = 6	30 min of in situ warm ischemia followed by 8 h cold ischemia preservation	2 h NMP with air ventilation	–
DMSO	N = 4	30 min of in situ warm ischemia followed by 8 h cold ischemia preservation	2 h NMP with air ventilation	DMSO
Rosiglitazone	N = 4	30 min of in situ warm ischemia followed by 8 h cold ischemia preservation	2 h NMP with air ventilation	Rosiglitazone
GW9662	N = 4	30 min of in situ warm ischemia followed by 8 h cold ischemia preservation	2 h NMP with air ventilation	GW9662

In the next step, in order to evaluate the effect of the selected targets on liver functional recovery from the above step, three more groups were designed: ④ Group Dimethylsulfoxide (DMSO, n = 4), DCD livers exposed to 30 min of in situ warm ischemia and 8 h cold ischemia preservation followed by 2 h air-oxygenated NMP with DMSO in the perfusate; ⑤ Group Rosiglitazone (n = 4), DCD livers exposed to 30 min of in situ warm ischemia and 8 h cold ischemia preservation followed by 2 h air-oxygenated NMP with the peroxisome proliferator activator receptor-γ (PPARγ)-specific agonist Rosiglitazone in the perfusate; and ⑥Group GW9662 (n = 4), DCD livers exposed to 30 min of in situ warm ischemia and 8 h cold ischemia preservation followed by 2 h air-oxygenated NMP with the PPARγ-specific antagonist GW9662 in the perfusate (presented in Table 1). The selective PPARγ agonist Rosiglitazone or PPARγ antagonist GW9962 was dissolved in DMSO and added in the perfusate with a final concentration of 10 μg/mL and 0.3 mg/kg, respectively^10,11^.

### Rat DCD model and liver NMP system

The rat DCD model and the ex situ liver perfusion system were modified from the previous studies^4,12^ and performed as published before^7^.

In brief, the DCD model was employed with induction of cardiac arrest due to incision of the diaphragm without prior heparinization in rats. The whole blood was withdrawn from the abdominal aorta in all the 30 rats. Blood harvest was performed in 2 min before induction of cardiac arrest in DCD rats or the sample harvest in control rats. The blood volume of each rat ranged from 12 to 15 ml. After 30 min of warm ischemia, livers were flushed in situ with 0~4 °C *heparin saline* through both the hepatic artery and portal vein*,* harvested and then preserved in 0~4 °C UW solution for 8 h before NMP.

The liver perfusion had perfusate comprised of rat red blood cells that recirculated through both hepatic artery and portal vein of liver grafts by means of a peristaltic pump (BT-100CA, Jieheng Pump Ltd, Chongqing, China). The perfusion medium, with the total volume of 36 mL, contained 24 ml whole heparinized blood supplemented with 10% sodium citrate, 1% penicillin and streptomycin, and 12 ml circuit priming solution with 45% lactated *ringer,* 5% *sodium* bicarbonate *and* 50% hydroxyethyl *starch.* The heat exchanger and bath thermostat maintained the temperature within the system at 38 °C. Liver grafts in the organ perfusion chamber were perfused for 2 h in a portal vein pressure-dependent manner with a constant portal vein perfusion pressure of 8–10 mmHg and hepatic artery pressure of 90–100 mmHg. The portal venous blood flow fluctuated between 5 and 15 ml/min, and the ratio between the portal vein flow and artery flow was 3:1. The oxygenator in the circulation of the hepatic artery was regulated with mechanical ventilation (Harvard Inspira Advanced Safety Ventilator, Pressure Controlled MAI 55-7059, Holliston, USA) and gassed with air or 100% oxygen through a hollow fiber dialyzer (SpectrumLabs, Rancho Dominguez, CA, USA). The oxygen concentration was adjusted to the blood gas value by oxygenator. The liver perfusion system was modified from the perfusion system^4,12^, and the same perfusion medium was used for two consecutive liver perfusions of 2 h each, supplemented with 4–5 ml circuit solutions after each perfusion to replenish the necessary loss.

### Sample collection and analysis

Perfusate samples for blood gas analysis were withdrawn from the cannula placed in the hepatic artery at the three-way stopcock and analyzed using an automatic blood gas analyzer (GEM Premier 4000, Instrumentation Laboratory Co., *Lexington,* USA). Perfusate samples were taken from the portal vein cannula for measurements of alanine aminotransferase (ALT) and aspartate aminotransferase (AST) using standard biochemical methods. The viability of the preserved livers was evaluated by changes in ALT (ΔALT = postperfusion ALT level – preperfusion ALT level) and AST (ΔAST = postperfusion AST level – preperfusion AST level) in the perfusate.

Liver biopsies obtained from the perfused liver were immediately snap-frozen with liquid nitrogen and stored at − 80 °C for the further detection of PPARγ and cytochrome P450 2E1 (CYP2E1), fixed with 10% buffered formalin for further morphological evaluation by performing hematoxylin/eosin (HE) staining, and kept at 0~4 °C for assessment of superoxide dismutase (SOD) activity, malondialdehyde (MDA) level and CYP2E1 activity, and tumor necrosis factor alpha (TNF-α) and interleukin-6 (IL-6) using an enzyme-linked immunosorbent assay (*ELISA*).

### Proteomics and metabolomics-based detection and its integrative analysis

The snap-frozen fresh liver tissues from control, hyperoxygenated NMP and air-oxygenated NMP groups were allocated for the simultaneous detection of mass spectrometry-based proteomics and metabolomics for detecting biomarker candidates.

Untargeted *proteomics*, protein identification and quantification by liquid chromatography-tandem mass spectrometry (LC–MS/MS) were performed as previously^13^. In brief, the peptides were enriched by prewashed antibody beads (PTM Biolabs, Hangzhou, China), followed by analysis with tandem-mass-tag labeling combined with LC–MS/MS on the tissue samples. The resulting MS/MS data were processed using the engine software MaxQuant (version 1.5.2.22, *Computational* *Systems* *Biochemistry,* Martinsried, *Germany*) and uploaded to Proteomics identifications database (PRIDE) under the project accession number PXD028641. A differentially expressed protein was considered significant if *P* < 0.05 and FOLD > 1.2.

Quantitative metabolomics was conducted on the XploreMET platform (Metabo-Profile Biotechnology, Shanghai, China) according to the previously published method^14^. Briefly, after the quality control, each specimen was introduced for GC-TOFMS analysis on a time-of-flight mass spectrometry (GC-TOF/MS) system (Pegasus) with an Agilent 7890B gas chromatograph (*Agilent* Technologies, Santa Clara, CA, *USA)*. The raw data from GC-TOF/MS were processed with XploreMET 3.0, and each data set was converted to comparable data for further statistical analysis (*P* < 0.05 and FOLD > 1.5 as significant difference).

The ratio of two adjacent metabolites from a known metabolic relation network was calculated for the analysis of individual metabolic alterations. The Z-score heat map was applied for data visualization of the entire relationship. A multivariate regression model with the O2PLS method and Kyoto Encyclopedia of Genes and Genomes (KEGG) analysis were applied to integrate proteomics data with metabolomics data. Integrative analysis and coregulated pathways by Spearman correlation analysis were constructed to elucidate the impact of oxygen concentration on DCD liver with NMP. Cytoscape/MetScape-based analysis was used to visualize and interpret the interaction network between proteins and metabolites.

### HE staining and morphological assessment of liver injury

Liver tissues were fixed in 4% paraformaldehyde in phosphate-buffered, embedded in paraffin wax, and stored at 4 °C. HE staining was performed, and histological analysis was evaluated according to Suzuki histological criteria by the pathologist as report before^15^.

### Western blot analysis

Protein extracts of liver tissue were prepared and separated by SDS–polyacrylamide gel electrophoresis (SDS-PAGE), transferred to polyvinylidene difluoride (PVDF) membrane by electroblotting, and processed for Western blot analysis as previously described^15^. The PVDF membranes were cut prior to hybridization with target-specific antibodies (PPARγ, CYP2E1) and the internal control, glyceraldehyde-3-phosphate dehydrogenase (GAPDH). Primary antibodies used were against PPARγ (1:1000, 16643-1-AP, Proteintech, Wuhan, China), CYP2E1 (1:1000, 19937-1-AP, Proteintech, Wuhan, China), and GAPDH (1:5000, 60004-1-Ig, Proteintech, Wuhan, China). Immunoreactivities were visualized by secondary horseradish peroxidase-conjugated rabbit antibody (1:2000, SA00001-2, Proteintech, Wuhan, China), mouse antibody (1:2000, SA00001-1, Proteintech, Wuhan, China), and the ECL Western Blotting Substrate (Solarbio Life Sciences, Beijing, China) according to the manufacturer's instruction of KPL Protein Detector Western Blot Kit (SeraCare Life Sciences, Milford, USA). The full-length membranes and the original blots are illustrated in the supplemental file.

### Fluorescent probes for selectively sensing activity

Based on the ratiometric fluorescent probes for selectively sensing activity of CYP2E1, liver S9 fraction was prepared from the perfused livers to detect levels of the P450-catalyzed drug metabolizing enzyme activities^16^.

### Determination of SOD activity and MDA level

Detection of the SOD activity at 550 nm and quantification of MDA content were performed using a WST-8 assay kit and thiobarbituric acid assay according to the manufacturer's instructions (S0131 and S0103, Beyotime Biotechnology, Haimen, *China*). Results were obtained using a Multiscan FC plate reader with SkanIt software (Thermo Fischer Scientific, Waltham, USA).

### ELISA

Inflammatory cytokine (TNF-α and IL-6) levels in the liver were measured according to the manufacturer's protocols (KE20001, Proteintech, Wuhan, China; EK0412, Boster Biological Technology, Wuhan, China).

### Statistical analysis

Values are given as means with a standard deviation (SD). Differences between the two groups were analyzed *by* using *two-tailed* unpaired *Student’*s *test, and differences* between more than two groups were analyzed *by* one-way analysis of variance (*ANOVA*), *and* Bonferroni test was used for a multiple comparison analysis*.* The statistical tests were employed by using SPSS version 21.0 (IBM, Armonk, New York, USA). A probability level of less than 5% (*P* < 0.05) was considered statistically significant.

### Ethics approval

The animal experiment was approved and supervised by the Animal Ethics Committee of Zhengzhou University, Zhengzhou, China (No.2019-KY-019).

## Results

### Air-oxygenated NMP confers a better functional recovery and a less inflammatory response of rat DCD liver after static cold preservation

Arterial oxygen pressures in oxygen-ventilated NMP and air-ventilated NMP were 576.3 ± 27.9 mmHg and 137.9 ± 17.6 mmHg with a significant difference (*t* = 13.307, *P* = 0.001). To compare the degree of liver recovery following air-ventilated NMP and oxygen-ventilated NMP, aminotransferases in perfusate, HE staining, SOD activity, MDA level and inflammation parameters of the perfused liver were investigated.

**Figure 1:**
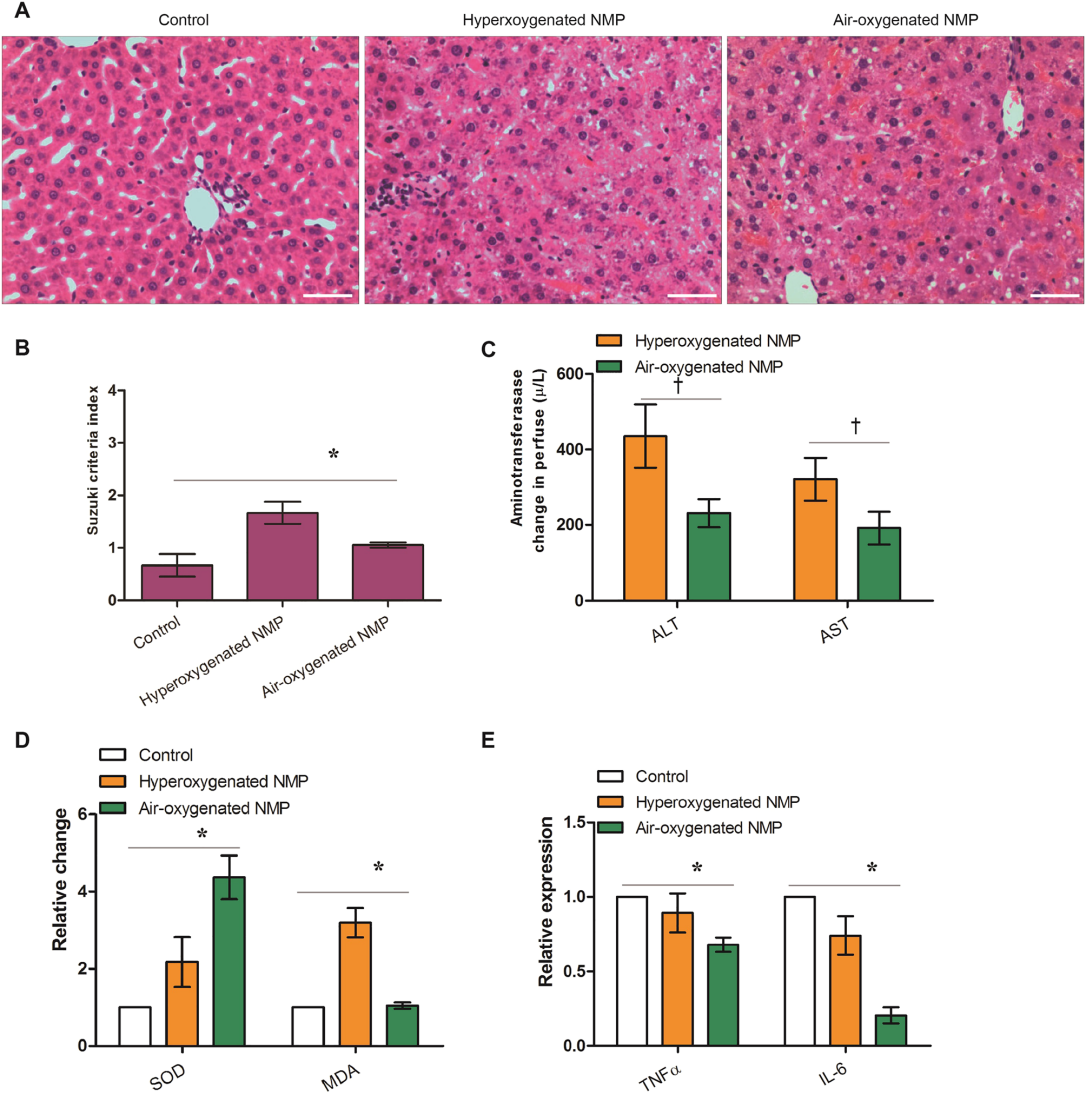
Air-oxygenated normothermic machine perfusion (NMP) confers a better functional recovery in rat liver from donation after cardiac death (*denotes significant differences among control, air-oxygenated NMP and hyperoxygenated NMP, *P* < 0.05; n = 6). (**A**) Histology (hematoxylin and eosin staining) of the perfused livers (hematoxylin counterstaining, original magnification × 200, and scale bars 50 μm); (**B**) uzuki sinusoidal injury score of the perfused livers on a scale from 0 to 4; (**C**) Changes in levels of ALT and AST in the NMP perfusate; (**D**) Relative changes in SOD activity and MDA level in the perfused livers; (**E**) Relative levels of inflammation factors, TNF-α and IL-6, in the liver performed using ELISA.

Histological staining showed that microvesicular steatosis with hepatocyte ballooning, inflammatory cell infiltration, sinusoidal dilatation and congestion indexed with Suzuki score in control, air-ventilated NMP and oxygen-ventilated NMP were significantly different (*F* = 25.800, *P* = 0.001) with a significant reduction following air-oxygenated NMP in comparison with that of hyperoxygenated NMP (0.750 ± 0.167 vs 1.583 ± 0.319) (*t* = 6.061, *P* = 0.001) (Fig. 1A,B).

The function parameters, ALT and AST levels in the perfusate showed an increasing trend during NMP. The elevations of ALT and AST following air-ventilated NMP were significantly lower than those of the NMP group (*t* = 4.925 and 2.985, *P* = 0.008 and 0.041) (Fig. 1C).

SOD activity and MDA level of liver tissues were 4.8 ± 2.1 U/mg and 8.4 ± 2.6 µmol/mg following oxygen-ventilated NMP, and 8.9 ± 2.2 U/mg and 2.7 ± 0.6 µmol/mg following air-ventilated NMP, respectively. SOD activity and MDA level in in control, air-ventilated NMP and oxygen-ventilated NMP were significantly different (*F* = 11.988 and 31.171, *P* = 0.008 and 0.001) with a significant difference following air-oxygenated NMP in comparison with that of hyperoxygenated NMP (*t* = 3.244 and 5.1527, *P* = 0.009 and 0.003) (Fig. 1D).

TNF-α and IL-6 in the liver following oxygen-ventilated NMP were 99.7 ± 12.1 pg/mg and 1217.3 ± 390.9 pg/mg, and TNF-α and IL-6 following air-ventilated NMP were 83.9 ± 8.5 pg/mg and 521.6 ± 221.2 pg/mg. TNF-α and IL-6 in in control, air-ventilated NMP and oxygen-ventilated NMP were significantly different (*F* = 37.194 and 15.635, *P* = 0.001 and 0.001) with a significant reduction following air-oxygenated NMP in comparison with that of hyperoxygenated NMP (*t* = 2.510 and 3.835, *P* = 0.031 and 0.003) (Fig. 1E).

### Integrative proteomics and metabolomics analysis reveals differentially expressed proteins and metabolites of the rat DCD liver between hyperoxygenated NMP and air-oxygenated NMP

**Figure 2:**
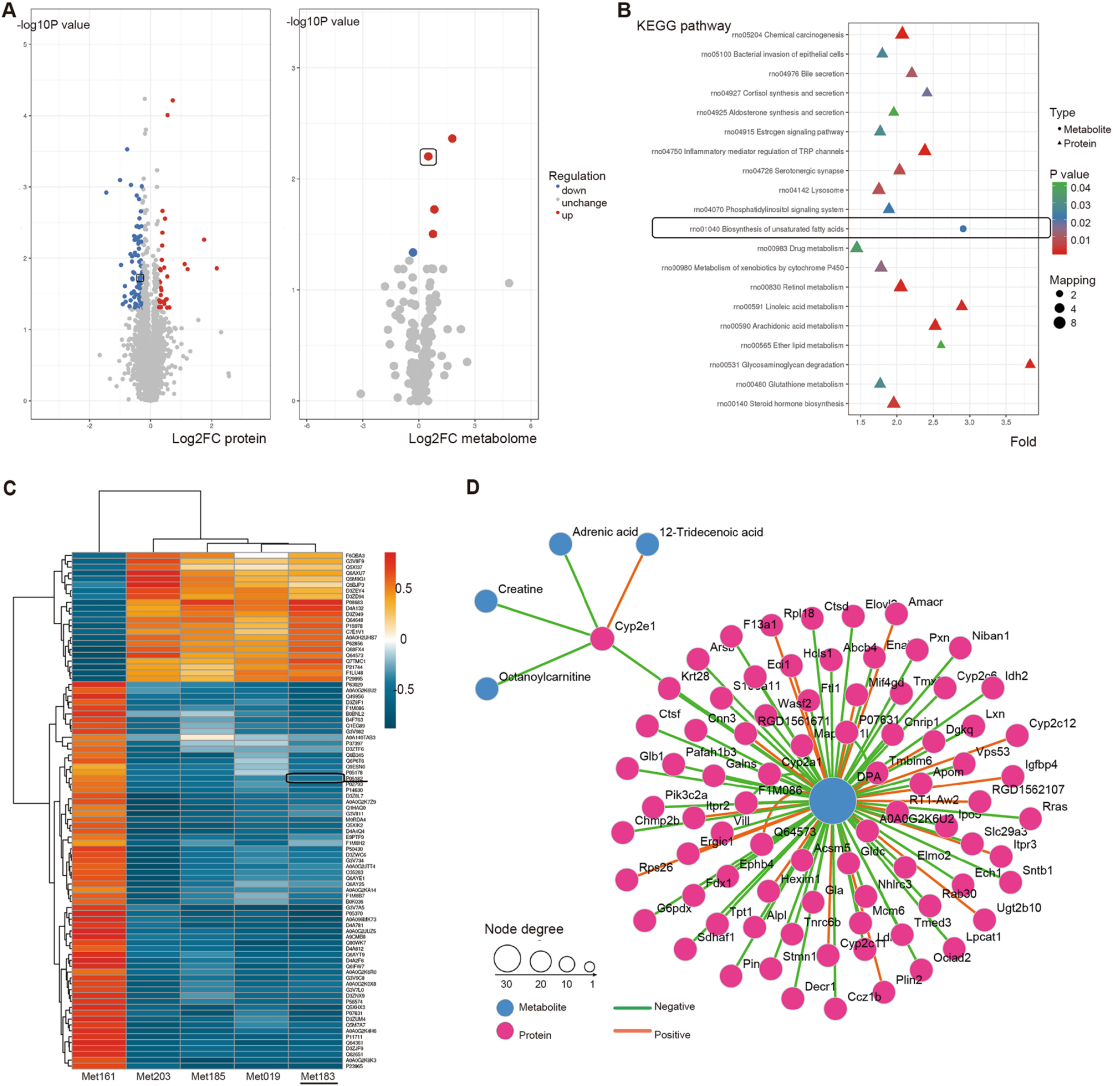
The integrative analysis *of proteomics* *and metabolomics*-based *detection* in rat livers from donation after cardiac death following static cold storage and normothermic machine perfusion (NMP). *Cyp2e1 represents* cytochrome P450 2E1 *protein;* DPA *represents* docosapentaenoic acid. (**A**) *The volcano plot* represents the results *of* the proteomics (left) and metabolomics (right) analysis *between* air-oxygenated and hyperoxygenated NMP. *Red spots* represent the upregulated proteins/metabolites; blue spots represent the downregulated proteins/metabolites; and grey spots represent the unchanged proteins/metabolites. The differentially expressed protein (CYP2E1) and metabolite (DPA) were identified and encircled. (**B**) The KEGG enrichment bubble chart shows the differentially expressed proteins (triangle) and metabolites (bubble) in the corresponding KEGG pathway. The color represents the degree of statistical significance. The size represents the number of the differentially changed proteins/metabolites in the corresponding pathways. The encircled pathways of biosynthesis of unsaturated fatty acids were identified to relate to CYP2E1 and DPA. (**C**) Heatmap of Spearman correlation analysis of significant proteins (y-axis) and metabolites (x-axis). Each color cell on the map corresponds to a Spearman correlation coefficient between the identified proteins (y-axis) and metabolites (x-axis). Red denotes positive correlation, whereas blue denotes negative correlation, and the dark color denotes the value of the Spearman correlation coefficient. The encircled pathway was shown as negatively correlated to relate with P05182 (CYP2E1) and Met183 (DPA). (**D**) Identified from proteomics and metabolomics, the protein-metabolite interaction network provides a visualization of the interaction between functionally related proteins indicated by the *red circle* and metabolites by the *blue circle. Cyp2e1 may react with metabolites (Octanoylcarnitine, Creatine, Adrenic acid, 12-Tridecenoic acid and DPA)*; DPA lies in the center of the reacted proteins including cyp2e1.

*The proteomics analysis identified 120 differentially expressed proteins with 33 upregulated proteins and 87 downregulated proteins* between hyperoxygenated NMP and air-oxygenated NMP (Fig. 2A)*. The metabolomics* analysis revealed that five *differential expressed metabolites* were identified (Fig. 2A).

Based on the data of the proteomics and metabolomics platforms, KEGG pathway analysis found that three differentially expressed proteins were highly correlated with the four identified metabolites. Among them, docosapentaenoic acid (DPA) and CYP2E1 were enriched in the pathway of biosynthesis of unsaturated fatty acids (Fig. 2B). A heatmap with two-dimensional hierarchical clustering was constructed to illustrate the correlation of significant proteins and metabolites by Spearman correlation analysis (Fig. 2C). In the heatmap, DPA was shown negatively correlated with the CYP2E1 protein (Fig. 2C). Cytoscape analysis was further used to visualize and interpret the interaction network between proteins and metabolites. The metabolite–protein interaction network provides a global view of potentially functional relationships between metabolites and connected proteins in Fig. 2D, where the correlation between DPA and CYP2E1 was highlighted.

### Intrahepatic changes of DPA, PPARγ, and CYP2E1 are associated with DCD liver functional recovery with NMP

**Figure 3:**
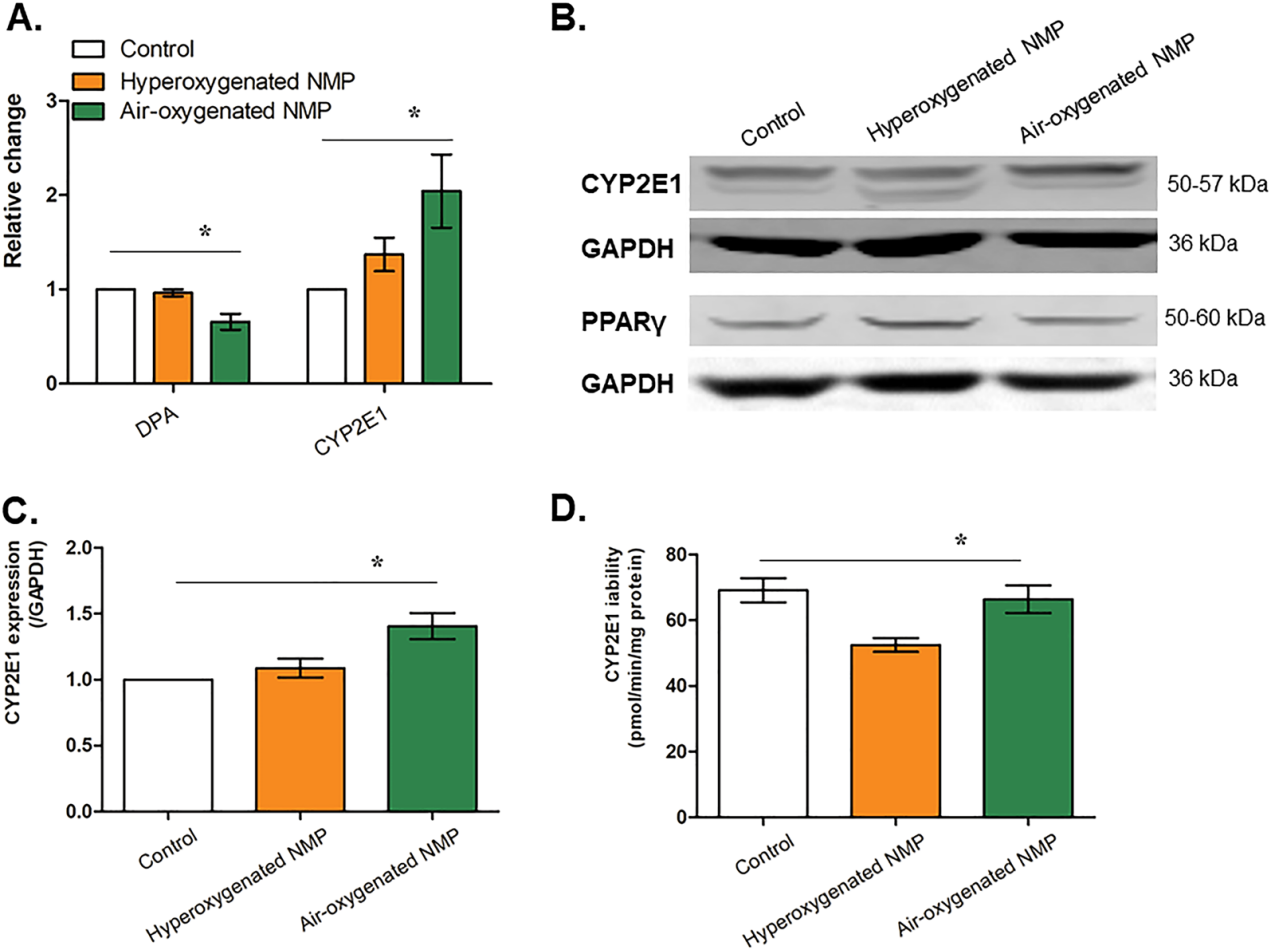
Expression of DPA, PPARγ and CYP2E1 in livers from donation after cardiac death using normothermic machine perfusion (NMP) (*denotes significant differences among control, hyperoxygenated NMP and air-oxygenated NMP, *P* < 0.05, n = 6). (**A**) Relative expression of DPA by metabolomics and CYP2E1 by proteomics; (**B**) Expressions of PPARγ and CYP2E1 by WB; (**C**) Semi-quantification of CYP2E1 by WB. (**D**) CYP2E1 activity quantified using the fluorescent probe.

The role of DPA has been confirmed as a PPARγ agonist in the previous study^17^. In addition, PPARγ agonists could reduce the expression and activity of CYP2E1^18^. In the current study, we detected the changes in DPA, PPARγ and CYP2E1 as well as the function recovery under air-ventilated NMP. In comparison with hyperoxygenated NMP, t*he quantitative metabolomics* analysis showed that DPA and CYP2E1 in control, air-ventilated NMP and oxygen-ventilated NMP were significantly different (*F* = 8.085 and 10.085, *P* = 0.010 and 0.001). DPA expression in the air-oxygenated NMP was downregulated by gas chromatography-mass spectroscopy (*t* = 3.776*, P* = 0.009, Fig. 3A), whereas the untargeted p*roteomics* analysis showed that CYP2E1 expression was upregulated (*t* = 2.839*, P* = 0.030, Fig. 3A). The expression of PPARγ and CYP2E1 were evidenced by Western blot, and CYP2E1 expression in control, air-ventilated NMP and oxygen-ventilated NMP was significantly different (*F* = 9.208, *P* = 0.001) with an increase in the air-oxygenated NMP in comparison with that of hyperoxygenated NMP (*t* = 2.613*, P* = 0.019, Fig. 3B,C).

The change in intrahepatic CYP2E1 activity was further detected by the fluorescent probe. The results showed that the CYP2E1 activity in control, air-ventilated NMP and oxygen-ventilated NMP were significantly different (*F* = 6.844, *P* = 0.008). CYP2E1 activity of air-oxygenated NMP with 66.438 ± 4.215 pmol/min/mg protein was higher than that of oxygen-oxygenated NMP with 52.479 ± 2.115 pmol/min/mg protein (*t* = 2.960*, P* = 0.014), and showed no significant difference with control with 69.180 ± 3.686 pmol/min/mg protein (*t* = 0.479*, P* = 0.643) (Fig. 3D). The above mentioned results demonstrated that the restoration of expression and activity of CYP2E1, due to downregulation of DPA and PPARγ^17,18^, was correlated with liver function, and therefore CYP2E1 might be indicated as a biomarker of DCD liver functional recovery during NMP.

### Targeting PPARγ might affect liver function of DCD liver with air-oxygenated NMP

The above findings demonstrated that DPA and PPARγ might participate in the liver functional recovery with NMP. PPARγ agonists have been shown to protect against liver injury^19,20^. Thus, during liver NMP, targeting PPARγ may theoretically improve DCD liver function.

**Figure 4:**
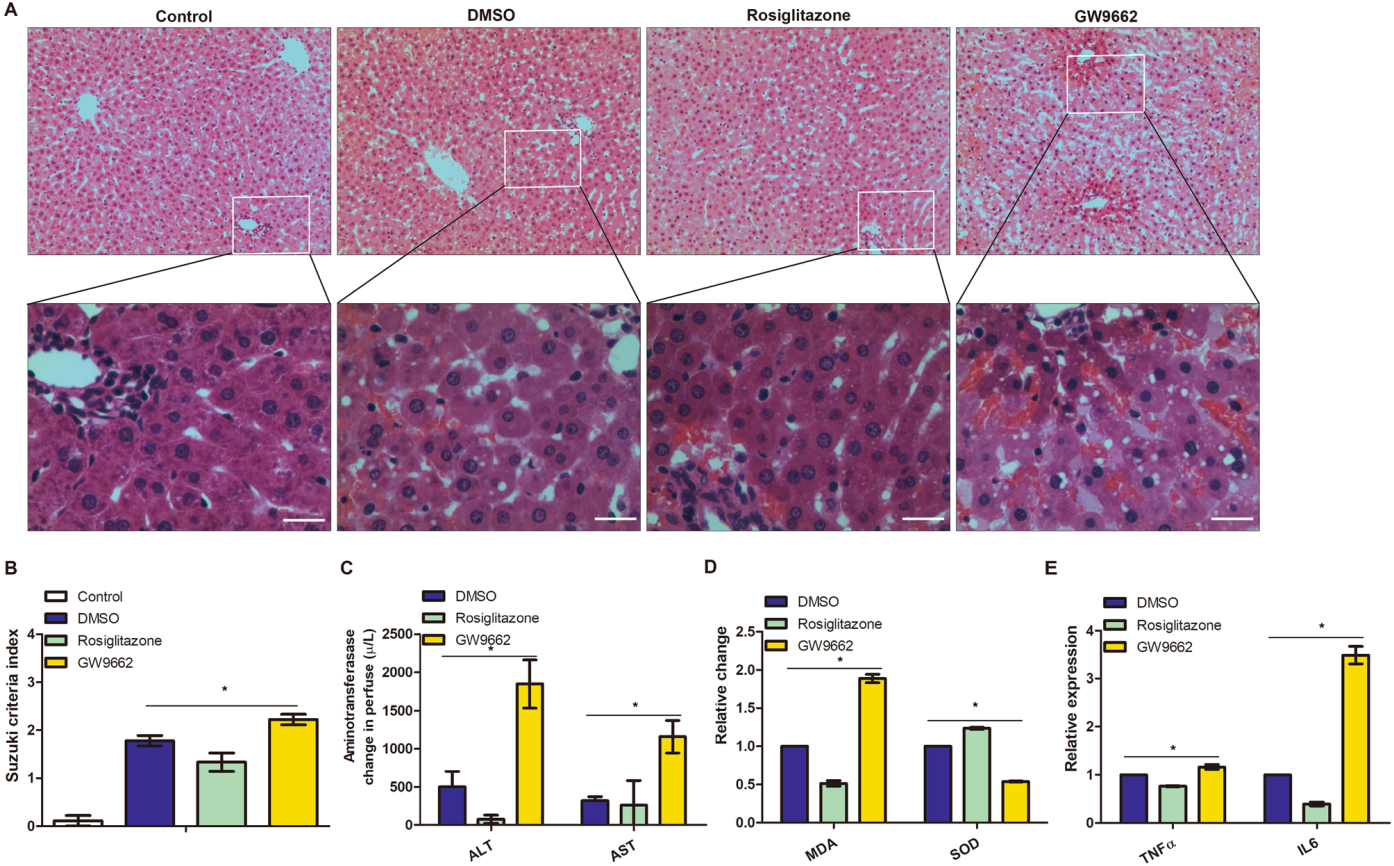
Effect of PPARγ on functions of livers from donation of cardiac death under air-oxygenated normothermic machine perfusion (NMP) (*denotes significant differences among DMSO, Rosiglitazone and GW6471, *P* < 0.05, n = 4). (**A**) Histology (haematoxylin and eosin staining, original magnification × 200, and scale bars 50 μm); (**B**) Suzuki sinusoidal injury scores of the perfused livers on a scale from 0 to 4; (**C**) Changes in levels of ALT and AST in NMP perfusate; (**D**) Relative changes in MDA level and SOD activity in the perfused livers; (**E**) Relative levels of TNF-α and IL-6 in the perfusate using ELISA.

To test the effect of PPARγ on DCD liver, administration of PPARγ agonist and antagonist in the perfusion circuit was employed in the air-oxygenated NMP system. In comparison with administration of DMSO and PPARγ agonist Rosiglitazone in the perfusion circuit, administration of PPARγ antagonist GW9962 worsened liver injury and inhibited functional recovery of DCD liver under NMP, as indicated by HE staining and Suzuki criteria score (*F* = 8.714, *P* = 0.017, Fig. 4A,B), ALT (*F* = 22.896, *P* = 0.002, Fig. 4C) and AST in the perfuse (*F* = 5.431, *P* = 0.004, Fig. 4C), MDA level and SOD activity (*F* = 5.990 and 6.894, *P* = 0.018 and 0.002, Fig. 4D) and TNF-α and IL-6 levels by ELISA (*F* = 6.426 and 12.602, *P* = 0.018 and 0.002, Fig. 4E); the multiple comparison analysis between PPARγ agonist or antagonist and the vehicle control (DMSO) demonstrated that PPARγ agonist via the NMP perfusion circuit benefits DCD liver functional recovery while PPARγ agonist acts in the opposite direction (*P* < 0.05).

## Discussion

In some experienced transplant centers, static cold preservation and NMP with subsequent transplantation are becoming the valid practice for DCD grafts^21^. NMP could simulate the physiological environment of cellular metabolism for liver recovery through oxygen delivery, and simultaneously NMP may initiate mitochondrial oxygenation, and trigger the cascade of inflammation and injury^4,5^. It remains unclear whether there is a consensus on the NMP protocol for DCD liver functional recovery.

After we compared the effect of NMP with air ventilation (PaO_2_ in the perfusate = 90~200 mmHg) and oxygen ventilation (PaO_2_ > 400 mmHg) on liver functional recovery in the rat DCD model^7^, the results demonstrated that air-ventilated NMP was associated with a superior liver morphological change, a better functional recovery and a less inflammatory response of the rat DCD liver with static cold preservation. The preclinical result of near-to-physiological oxygenated NMP offered a better way to expand clinical usage of the *extended* *criteria* *donor liver*.

Based on the above findings, the *proteomics* *and* *metabolomics* detections were used to investigate the mechanism of air-ventilated NMP on DCD liver with the rat NMP model^7^. The integrative *proteomics* *and* *metabolomics* analysis revealed that the downexpressed DPA and upregulated CYP2E1 were functionally correlated with liver functional recovery with air-ventilated NMP. The function of the metabolite DPA has been confirmed as a PPARγ agonist in the previous study^17^. *In addition,* CYP2E1 has been shown to be transcriptionally and functionally linked to PPARγ and liver function, and PPARγ agonist can reduce CYP2E1 expression and activity^18^, potentially protecting in liver injury^19,20^. Thus, air-ventilated NMP benefits DCD liver functional recovery, which might be mediated through the DPA-PPARγ axis. Furthermore, in our present study, PPARγ agonist Rosiglitazone improved liver function in the ex vivo liver NMP system whereas GW9962 functioned the opposite, indicating that PPARγ could be regarded as a therapeutic target against liver injury from DCD.

Meanwhile, the *proteomics* *and* *metabolomics* detections were performed to identify *the differentially expressed proteins and metabolites* between hyperoxygenated NMP and air-oxygenated NMP. These targets may provide more sensitive biomarkers to assess the liver real-time viability, and thus help to make more precise judgement on use of DCD liver graft during NMP after graft retrieval. To assess the graft viability of perfused livers for transplantation, a couple of perfusate parameters and bile parameters before implantation have been developed^21^. Expression and activity of liver cytochrome P450 isozymes were significantly changed after ischemia/reperfusion injury, indicating their use as biomarkers of function recovery during liver injury^22,23^. Cytochrome P450 1A2 was recently developed as a biomarker of liver function assessment during NMP, while the mechanism is unclear^24^. CYP2E1 is part of the cytochrome P450 family of drug-metabolizing enzymes in the liver. The CYP2E1 protein level was positively associated with high inflammation scores through ROS-induced lipid peroxidation^25,26^. Notably, CYP2E1 activities and concentration were gradually decreased during cold preservation and reperfusion^27^. Our research group constructed the ratiometric fluorescent probes for selectively sensing the activity of cytochrome P450, including CYP2E1, 1A2, 2J2, 3A4 and 3A5^16,28,29^. In the present study, DCD liver functional recovery with air-ventilated NMP was found to be correlated with the upregulated expression and activity of CYP2E1. Thus, intrahepatic expression and activity of CYP2E1 would be indicated as a biomarker to monitor DCD liver graft function during NMP. In addition, the fluorescence probe is an easy and feasible method to evaluate the intrahepatic CYP2E1 activity and DCD liver functional recovery*.*

There are still some limitations to the study should be kept in mind. Firstly, we selected the perfusion period of 120 min according to the most used animal study and clinical practices. The longer-term perfusion, which may further improve perfusion effect^30^, was not employed in the current study. Another consideration is the selection of the liver functional parameters. With the limitation of the size of the research animal and the detection, some biological parameters, including lactate clearance and bile production^21,31^, were not included and compared. Additionally, results from the ex vivo reperfusion model could represent preliminary data and still require confirmation in appropriate transplantation models in future studies.

## Conclusions

The results of the present study showed that air-ventilated NMP can simulate a near-to-physical oxygen pressure through artery perfusion, and thus confer better liver functional recovery from DCD rats following ischemia–reperfusion injury. The integrative *proteomics* *and* *metabolomics* analysis and the further functional tests confirmed that the DAP (PPARγ agonist)-PPARγ-CYP2E1 axis might provide promising biomarkers and therapeutic targets, and thus predict and improve liver graft quality from DCD donors.

## Supplementary Information

Supplementary Information.

## Data Availability

The data will be available on reasonable request from the corresponding authors.

## Abbreviations

CYP2E1: Cytochrome P450 2E1
DCD: Donation after cardiac death
DMSO: Dimethylsulfoxide
DPA: Docosapentaenoic acid
HE: Hematoxylin/eosin
HMP: Hypothermic machine perfusion
IHC: Immunohistochemistry
IL-6: Interleukin-6
MDA: Malondialdehyde
NMP: Normothermic machine perfusion
PPARγ: Peroxisome proliferator activator receptor-γ
SCS: Static cold storage
SOD: Superoxide dismutase
TNF-α: Tumor necrosis factor alpha
