# Supplementary material for: Cytochrome P450 2E1 predicts liver functional recovery from donation after circulatory death using air-ventilated normothermic machine perfusion
Source: Sci Rep. 2022 May 6;12:7446. doi: 10.1038/s41598-022-11434-y (PMC9076671; doi:10.1038/s41598-022-11434-y)

Repeat 1

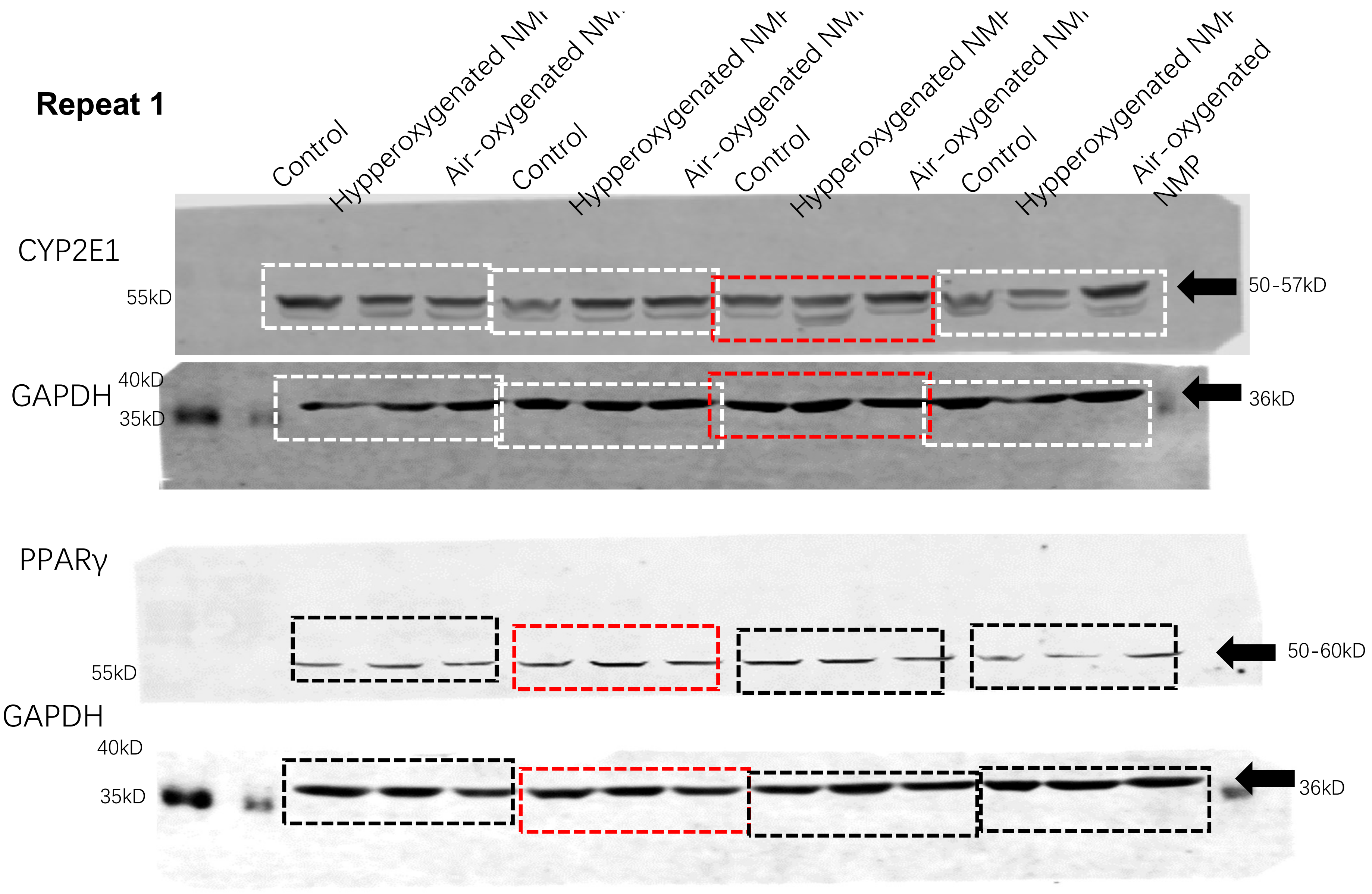

Control
Hypperoxygenated NMP
Air-oxygenated NMP
Control
Hypperoxygenated NMP
Air-oxygenated NMP
Control
Hypperoxygenated NMP
Air-oxygenated NMP
Control
Hypperoxygenated NMP
Air-oxygenated NMP
CYP2E1
55kD
50-57kD
GAPDH
40kD
35kD
36kD
PPARγ
55kD
50-60kD
GAPDH
40kD
35kD
36kD

Repeat 2

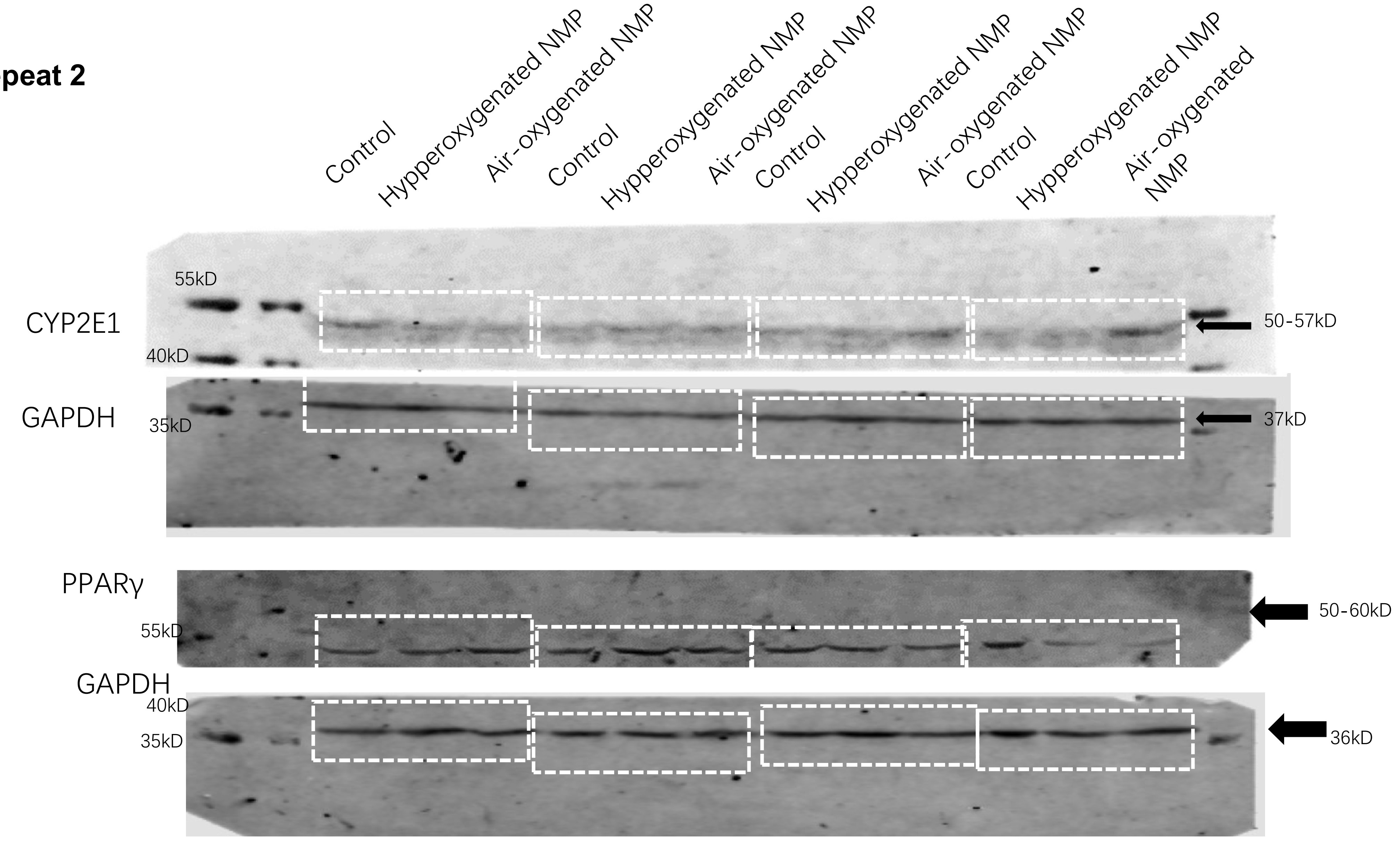

Control
Hypperoxygenated NMP
Air-oxygenated NMP
Control
Hypperoxygenated NMP
Air-oxygenated NMP
Control
Hypperoxygenated NMP
Air-oxygenated NMP
Control
Hypperoxygenated NMP
Air-oxygenated NMP
CYP2E1
55kD
40kD
50-57kD
GAPDH
35kD
37kD
PPARγ
55kD
50-60kD
GAPDH
40kD
35kD
36kD

Repeat 3

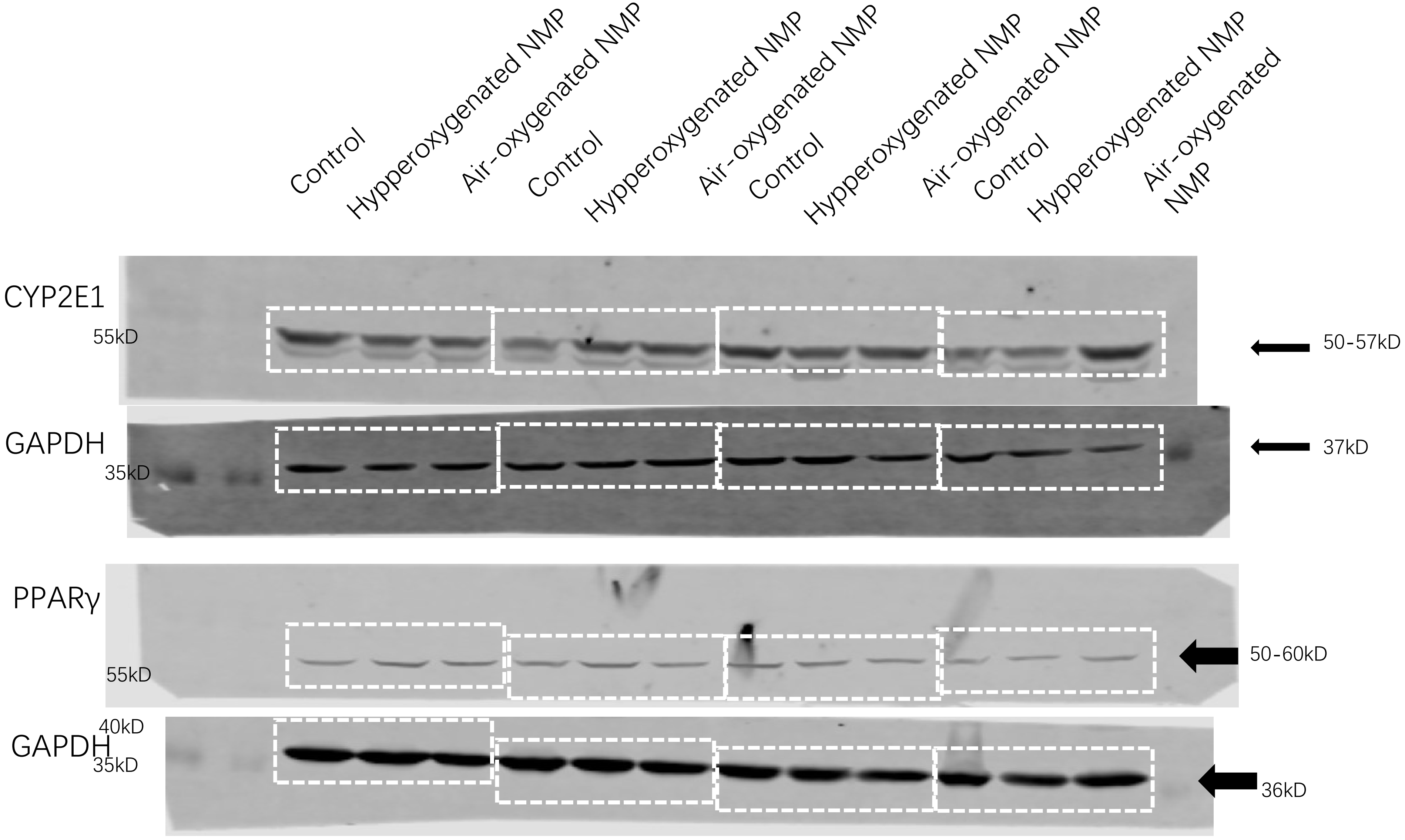

Control
Hypperoxygenated NMP
Air-oxygenated NMP
Control
Hypperoxygenated NMP
Air-oxygenated NMP
Control
Hypperoxygenated NMP
Air-oxygenated NMP
Control
Hypperoxygenated NMP
Air-oxygenated NMP
CYP2E1
55kD
50-57kD
GAPDH
35kD
37kD
PPARγ
55kD
50-60kD
GAPDH
40kD
35kD
36kD

Full-length membrane

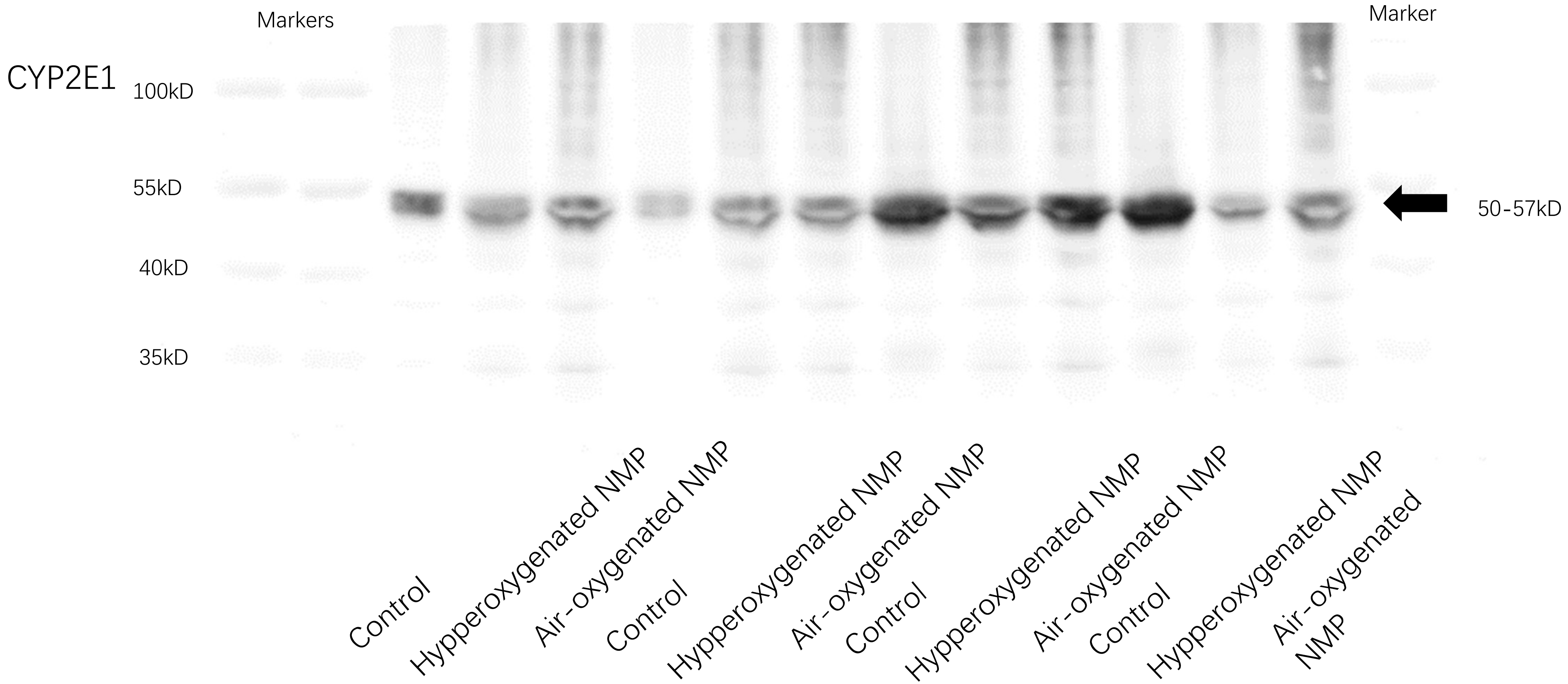

Full-length membrane

PPARγ

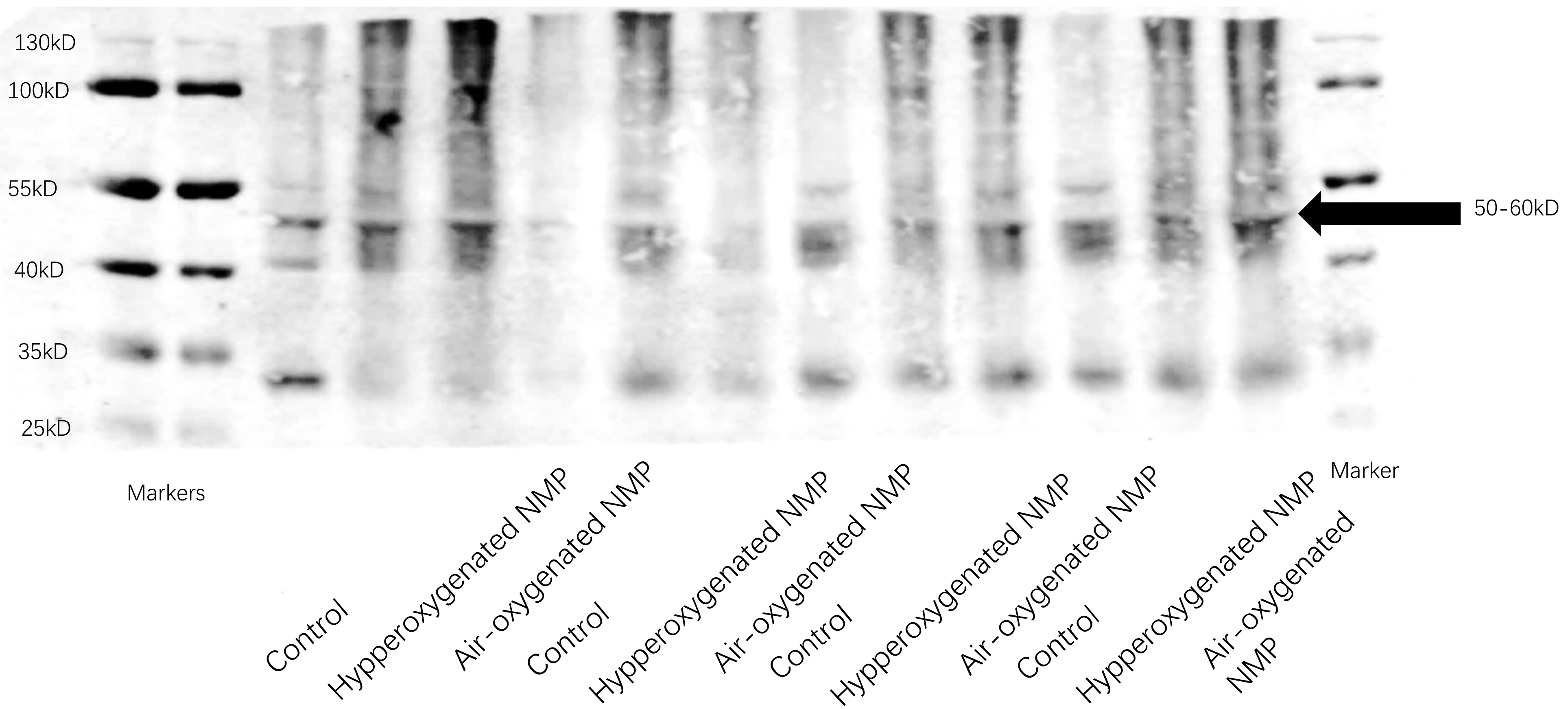

Supplement: Supplementary file 1 — Supplementary Information. [file 41598_2022_11434_MOESM1_ESM.pdf]
